# TNFR2 ligation in human T regulatory cells enhances IL2-induced cell proliferation through the non-canonical NF-κB pathway

**DOI:** 10.1038/s41598-018-30621-4

**Published:** 2018-08-13

**Authors:** Jun Wang, Ricardo Ferreira, Wanhua Lu, Samatha Farrow, Kate Downes, Lutz Jermutus, Ralph Minter, Rafia S. Al-Lamki, Jordan S. Pober, John R. Bradley

**Affiliations:** 1 Department of Medicine, NIHR Cambridge Biomedical Research Centre, University of Cambridge, Cambridge, United Kingdom; 2 JDRF/Wellcome Diabetes and Inflammation Laboratory, Wellcome Centre for Human Genetics, University of Oxford, Oxford, UK; 3 Department of Haematology, University of Cambridge, Cambridge, UK; 4 MedImmune Ltd., Granta Park, Cambridge, CB21 6GH UK; 5 Department of Immunobiology, Yale University School of Medicine, New Haven, CT United States

## Abstract

Human T regulatory cells (T regs) express high levels of TNF receptor 2 (TNFR2). Ligation of TNFR2 with TNF, which can recognise both TNFR1 and TNFR2, or with a TNFR2-selective binding molecule, DARPin 18 (D18) activates canonical NF-κB signalling, assessed by IκBα degradation, and the magnitude of the response correlates with the level of TNFR2 expression. RNA-seq analysis of TNF- or D18-treated human T regs revealed that TNFR2 ligation induces transcription of *NFKB2* and *RELB*, encoding proteins that form the non-canonical NF-κB transcription factor. In combination with IL2, D18 treatment is specific for T regs in (1) stabilising NF-κB-inducing kinase protein, the activator of non-canonical NF-κB signalling, (2) inducing translocation of RelB from cytosol to nucleus, (3) increasing cell cycle entry, and (4) increasing cell numbers. However, the regulatory function of the expanded T regs is unaltered. Inhibition of RelB nuclear translocation blocks the proliferative response. We conclude that ligation of TNFR2 by D18 enhances IL2-induced T regs proliferation and expansion in cell number through the non-canonical NF-κB pathway.

## Introduction

T regulatory cells (T regs), characterised by expression of the Forkhead family transcription factor Foxp3 (forkhead box P3), are key regulators of the immune response^1^. Tumour necrosis factor-α (TNF) is a pleiotropic cytokine critical in both innate and adaptive immunity, which signals through two cell surface receptors, TNFR1 and TNFR2. TNF has been variably reported to either promote^2–4^, inhibit^5–7^, or have no effect on T regs function^8^. Whilst TNFR1 is the dominant receptor for pro-inflammatory responses^9,10^, human and murine T regs express high levels of TNFR2^11,12^. We hypothesized that TNFR2 signalling, which is preferentially activated by “membrane” TNF expressed on the surface of another cell, could have distinct effects on T regs that may be obscured by TNFR1 signals.

Although T regs were initially thought to be relatively inert in cell surface signal transduction^13^, reduced signal transduction of T regs compared to conventional T effector cells (T cons) appears to be limited to TCR engagement^14^. In fact, interleukin-2 (IL2) or IL6- induced phosphorylation of signal transducer and activator of transcription 5 (STAT5) is higher in T regs compared to T cons^14^. NF-κB-dependent gene transcription is activated by nuclear translocation of homodimers or heterodimers of the DNA binding RelA (p65), RelB, c-Rel, NFκB1 (p50), and NFκB2 (p52). Canonical NF-κB-mediated transcription most often involves p50/RelA heterodimers that are normally held in the cytoplasm through association with inhibitory IκB proteins. Target gene transcription is initiated in response to binding of a ligand, such as TNF, to its cell surface receptor, triggering activation of IκB kinase (IKK)β, followed by phosphorylation, ubiquitination and degradation of IκBα and releasing and allowing nuclear translocation of p50/RelA. Non-canonical NF-κB signalling is activated by a different set of receptors, resulting in a signal that stabilizes NFκB inducing kinase (NIK) which activates IKKα leading to phosphorylation, ubiquitination and partial degradation of p100NFκB2 to generate p52NFκB2 followed by translocation of RelB/p52NFκB2 complexes into the nucleus^15^. The set of genes targeted by canonical and non-canonical NF-κB-mediated transcription may differ.

We recently reported that a TNFR2-specific binding molecule, designated DARPin 18 (D18, also named as TREG005) induced IκBα degradation in human T regs, indicative of canonical NF-κB signalling^16^. In this study, we have investigated further responses of human T regs isolated from whole blood of healthy human volunteers to TNF or D18 using RNA-seq, immunoblotting and immunofluorescence. We find that in T regs D18 initially uses canonical NF-κB signalling to prime and then activate non-canonical NF-κB signalling and that the latter pathway enhances the proliferative response of T regs to IL2.

## Results

### D18 preferentially induces IκBα degradation in T regs

**Figure 1:**
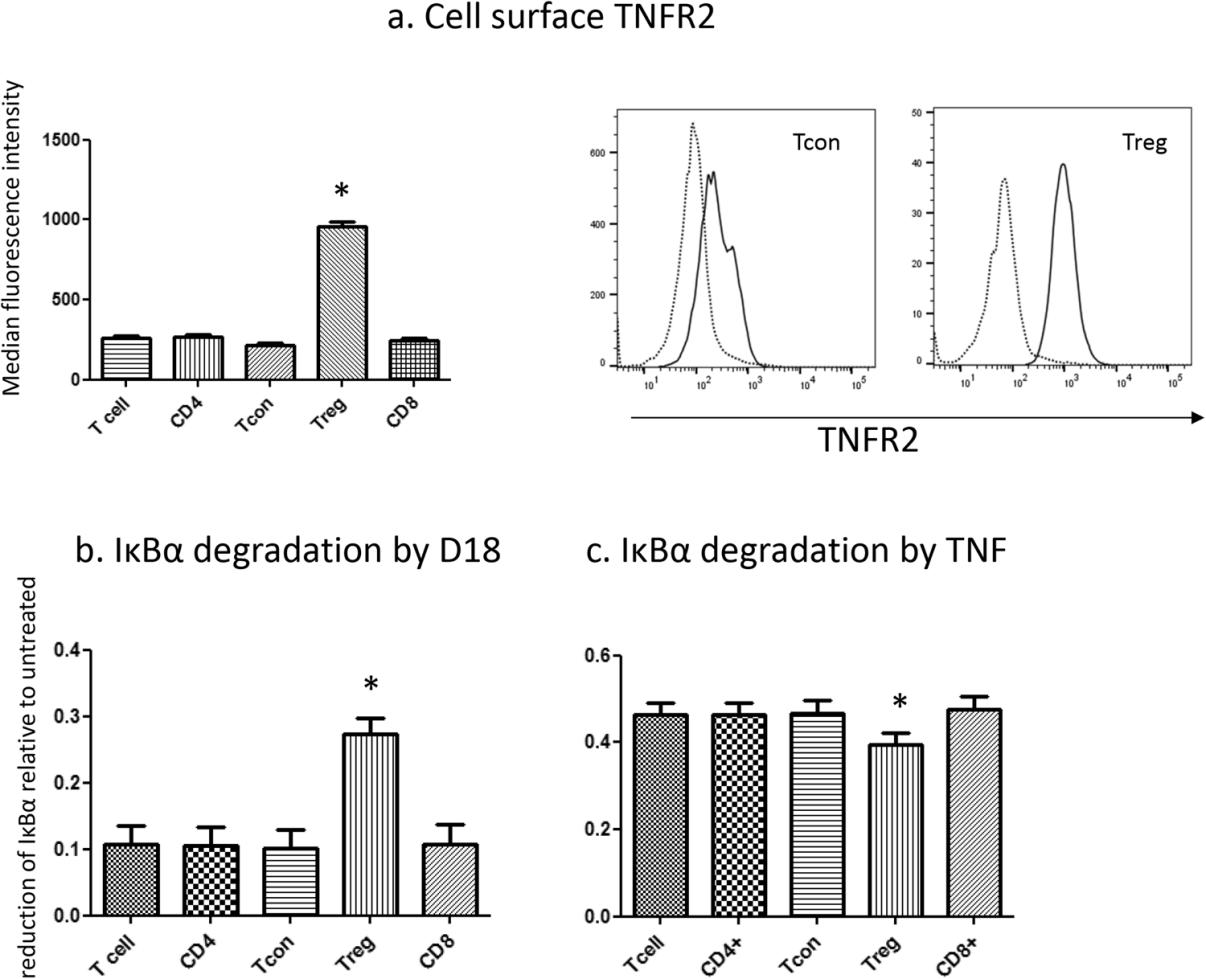
Surface TNFR2 level on different subset of T cells and their response to D18 and TNF. Peripheral lymphocytes from 24 donors were analysed with flow cytometer and cell phenotype was gated as described in the Methods. (**a**) Level of cell surface TNFR2 immunostained with two different monoclonal antibodies showed similar result. TNFR2 in T regs (CD4^+^CD25^hi^CD127^lo^) was 3 times higher than T cons (CD4^+^CD25^lo^CD127^hi^) (959 ± 24 vs. 218 ± 13; p < 0.0001). CD4^+^ T cell and CD8^+^ T cell population in general showed similar level of TNFR2 compared to the T cell population (TCR^+^CD56^−^) analysed as a whole. Typical histograms showed the cell surface TNFR2 in T cons and T regs (dotted line: isotype control antibody; solid line: anti-TNFR2 antibody). (**b**) IκBα degradation induced by D18 was 27.4 ± 2.4% in T regs, compared to 10.2 ± 2.7% in T cons (P < 0.0001). CD4^+^ T cell and CD8^+^ T cell population in general showed a similar degree of IκBα degradation (10.5 ± 2.7% and 10.6 ± 2.9%) compared to the T cell population analysed as a whole (10.8 ± 2.6%). (**c**) IκBα degradation induced by TNF was 39.6 ± 2.5% in T regs, compared to 46.8 ± 2.7% in T cons (P < 0.0001). CD4^+^ T cell and CD8^+^ T cell population in general showed similar degree of IκBα degradation (46.3 ± 2.6% and 47.7 ± 2.7%) compared to the T cell population analysed as a whole (46.4 ± 2.6%). (*p < 0.05 compared to any other group).

**Figure 2:**
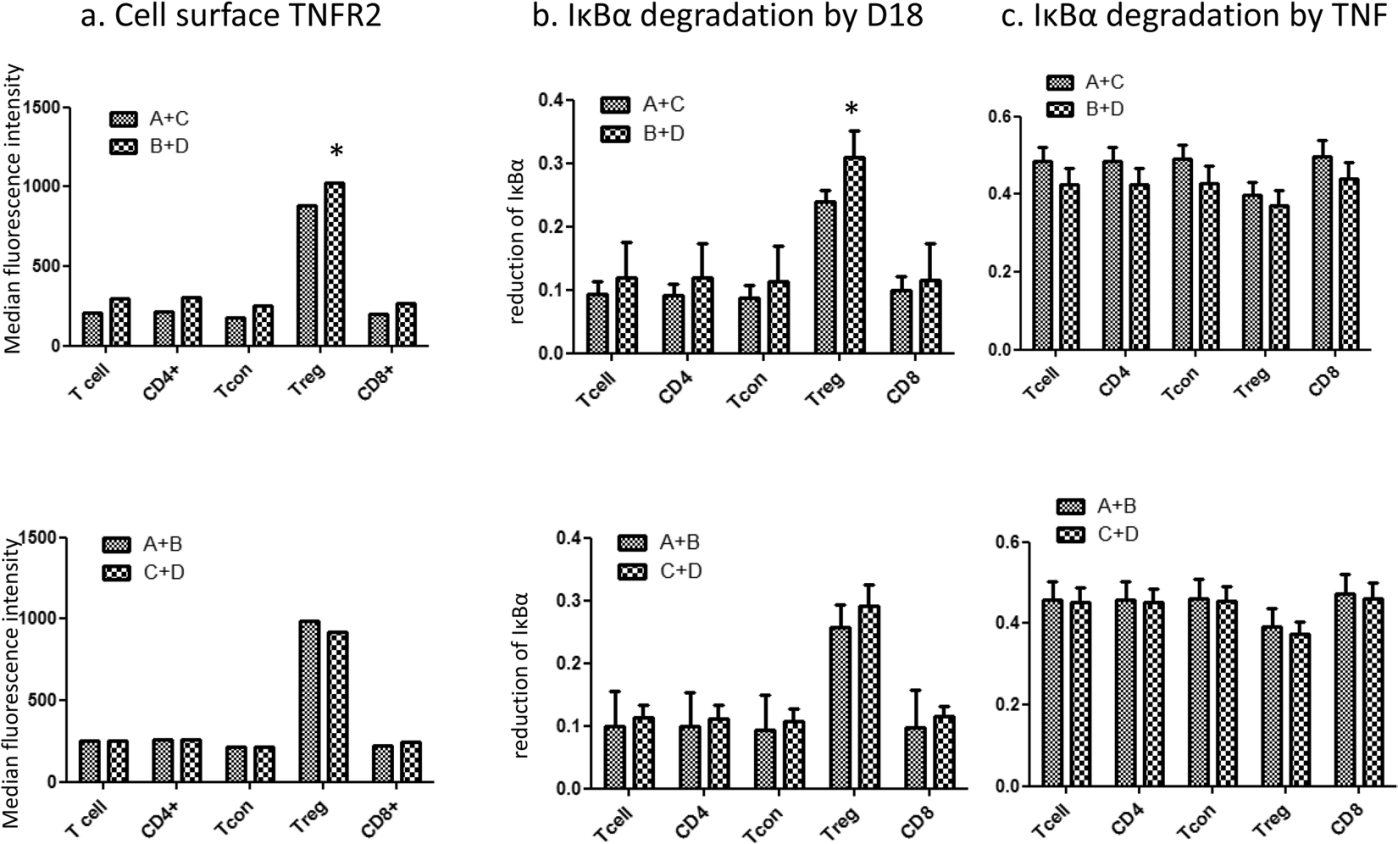
D18 induced more IκBα degradation in T regs that express higher level of cell surface TNFR2. (**a**) Cell surface TNFR2 expressed by 12 donors from group A plus group C on average (in arbitrary units) was 881 ± 40, significantly lower (p < 0.0001) compared to 1027 ± 47 from the 12 donors from group B plus group D. The recombination of 12 donors from group A plus group B showed surface level of TNFR2 as 985 ± 29, which showed no significant difference compared to 12 donors from group C plus group D as 923 ± 60. T cons, CD4^+^ T cell and CD8^+^ T cell and the T cell population as a whole showed similar pattern. (**b**) D18 induced more IκBα degradation in T regs from the 12 donors of group B + D than the 12 donors of group A + C (29 ± 3% vs. 23 ± 1%, p = 0.040). The recombination group A + B and B + D showed no difference in IκBα degradation (26 ± 3% vs.29 ± 3%, p = 0.470). Comparison between other phenotypic groups showed no significant difference. (**c**) TNF induced IκBα degradation in T regs from the 12 donors of group B + D was 36 ± 3%, and although less than the average 39 ± 3% for the 12 donors of group A + C the difference was not significant. Comparison between other groups showed no significant difference. (*p < 0.05 compared to the other group of T regs.)

PBMCs derived from human blood from 24 healthy adult volunteers were analysed for TNFR2 expression within 4 h after venesection. Cells were gated as in Supplement figure 1. The average level of cell surface TNFR2 was three times higher on T regs compared to T cons, or total CD4^+^ or CD8^+^ T cells (Fig. 1a). We have previously reported that the amount of IκBα degradation was related to the level of cell surface receptors upon saturated ligand binding^10^. Consistent with our previous report, D18 induced significantly more IκBα degradation in T regs compared with T cons or total CD4^+^ or CD8^+^ T cells (Fig. 1b), and the difference was proportional to the level of TNFR2 expression. In contrast, treatment with recombinant human TNF, which engages both TNFR1 and TNFR2, caused more extensive IκBα degradation in all groups of peripheral blood T cells but was more active on T cons, including both the CD4^+^ and CD8^+^ subsets, than in T regs (Fig. 1c). To determine whether the higher level of IκBα degradation was causally related to the higher level of cell surface TNFR2 expression, we first assessed whether single nucleotide polymorphisms in the promoter region (rs522807)^17^ or reading frame (rs1061622)^18^ of the *TNFR2* gene altered cell surface expression of TNFR2 in T regs from 24 volunteers recruited from the NIHR Cambridge BioResource. T regs from volunteers with genotype rs 522807 (C, C n = 12) express higher TNFR2 than (A, C n = 12), while the level of expression was the same between genotype rs1061622 (T, T n = 12) and (G, G n = 12) (Table 1 and Fig. 2a). Using these cells, we found that D18 induced significantly more IκBα degradation in T regs from volunteers with rs 522807 C,C and thus expressed higher levels of cell surface TNFR2 (Fig. 2b) than in volunteers with the other genotype. No significant differences in the level of IκBα degradation was observed among different groups in response to TNF (Fig. 2c).

**Table 1:** 24 donors were divided into 4 groups according to two of their TNFR2 SNPs.

Group	Genotype
A	rs1061622 (T, T), rs 522807 (A, C)
B	rs1061622 (T, T), rs 522807 (C, C)
C	rs1061622 (G, G), rs 522807 (A, C)
D	rs1061622 (G, G), rs 522807 (C, C)

### D18 and TNF induce differential transcriptome regulation in T regs

CD4^+^CD25^+^CD127^−^ T regs of more than 87% purity, isolated from 3 samples of human PBMCs, were treated with D18 or TNF. Total RNA (250 ng) from each sample was analysed by stranded mRNA-Seq. We used the edgeR linear model approach with a paired-sample design to make pairwise comparisons between untreated and TNF or D18-treated cells in each treatment group. 3 of the top 4 genes regulated by D18 were the same as those regulated by TNF (Tables 2 and 3). As expected *NFKB1A* (encoding IκBα) was upregulated as demonstrated in our flow cytometry data (Figs 1 and 2). However, the presence of *NFKB2* and *RELB*, two critical genes in the non-canonical NF-κB pathway, was unexpected.

**Table 2:** Top 4 genes regulated by D18 in Tregs.

GeneName	logFC	logCPM	PValue	FDR
NFKB2	1.07	5.64	1.73E-29	3.07E-25
MIAT	0.75	6.25	3.55E-17	3.14E-13
RELB	1.48	4.51	1.07E-13	6.30E-10
NFKBIA	0.64	6.95	1.45E-12	6.42E-09

FC: Fold change; CPM: counts per million reads.

**Table 3:** Top 4 genes regulated by TNF in T regs.

GeneName	logFC	logCPM	PValue	FDR
RELB	2.87	5.67	4.99E-81	8.79E-77
NFKB2	2.17	6.48	1.67E-34	5.89E-31
NFKBIA	1.78	7.73	1.03E-32	3.02E-29
N4BP3	1.69	2.68	1.91E-11	5.61E-09

**Figure 3:**
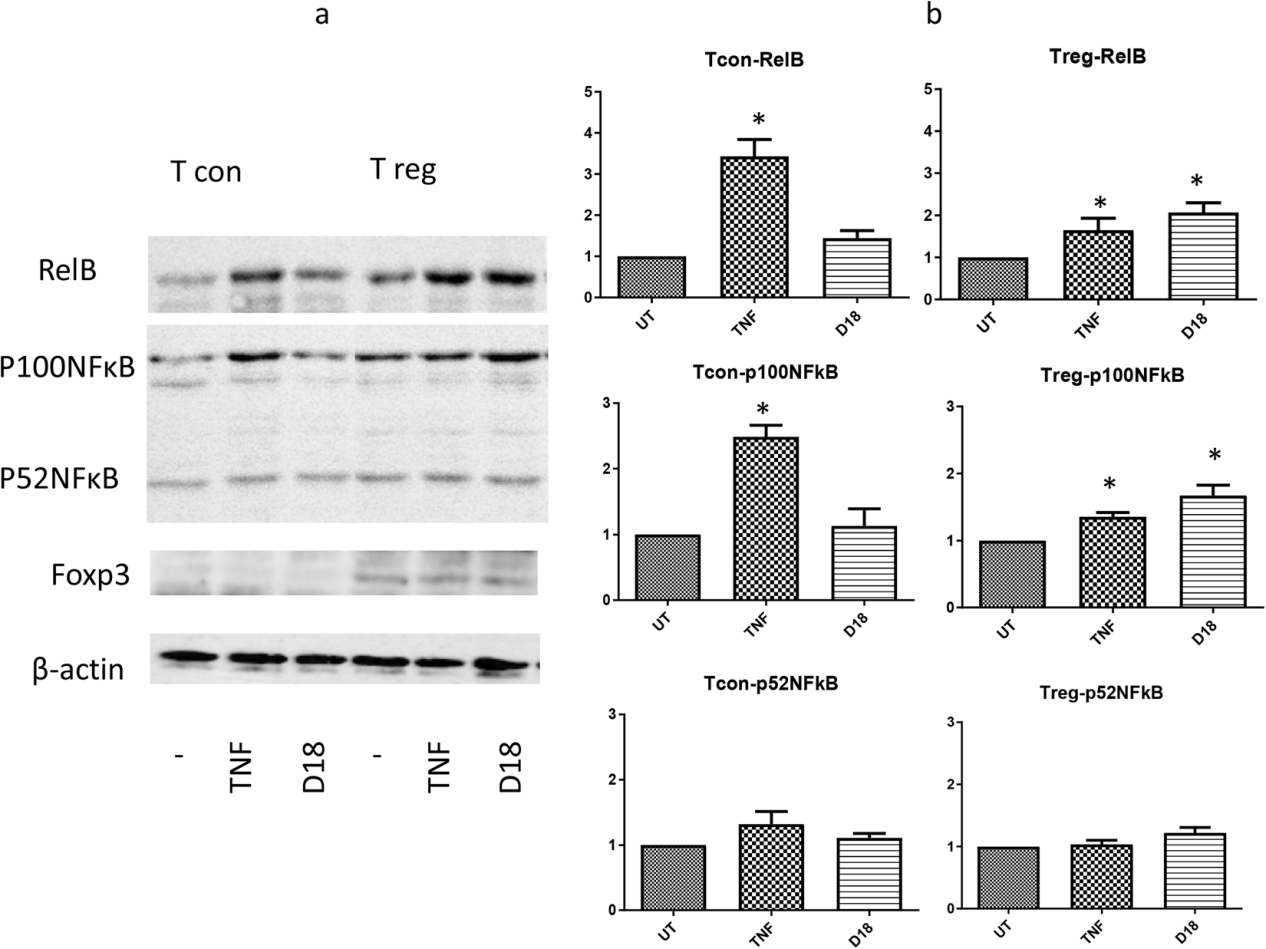
D18 up-regulated RelB and p100NFκB protein expression only in T regs. T cons and T regs were treated with TNF or D18 alone for 8 hours as indicated in the method. (**a**) Representative immunoblots of RelB, p100/p52NFκB, Foxp3 and actin are shown from 3 experiments cropped from the same gel (supplement figure 2). Foxp3 was only detected in T regs, and not regulated by any treatment. (**b**) TNF significantly up-regulated RelB and p100NFκB protein in both T cons and T regs (T cons: p = 0.028, p = 0.013; T regs: p = 0.048, p = 0.040), with a more prominent effect on T cons; D18 only significantly up-regulated RelB and p100NFκB in T regs (p = 0.048, p = 0.046), not in T cons. p52NFκB was not significantly regulated by any treatment. (*p < 0.05 compared to the untreated.)

To examine if these differences in transcripts correlated with protein expression, we immunoblotted RelB and NFκB2 (p100/p52NFκB). As shown in Fig. 3, TNF significantly upregulated RelB in both T cons and T regs, while D18 significantly upregulated RelB only in Foxp3 positive T regs. The p100 form of NFκB2 protein showed similar results as RelB protein, although the p52 processed form of NFκB2 showed no significant difference between the treated and untreated groups. This regulation at the protein level confirmed our RNA seq findings in that D18 regulated NF-κB genes preferentially in T regs, whereas it had no effect on the cell lineage marker Foxp3 (Supplement tables 1 and 2).

### D18 synergizes with IL2 to upregulate NFκB2 and Ki67 expression and activate the non-canonical NF-κB pathway in T regs

**Figure 4:**
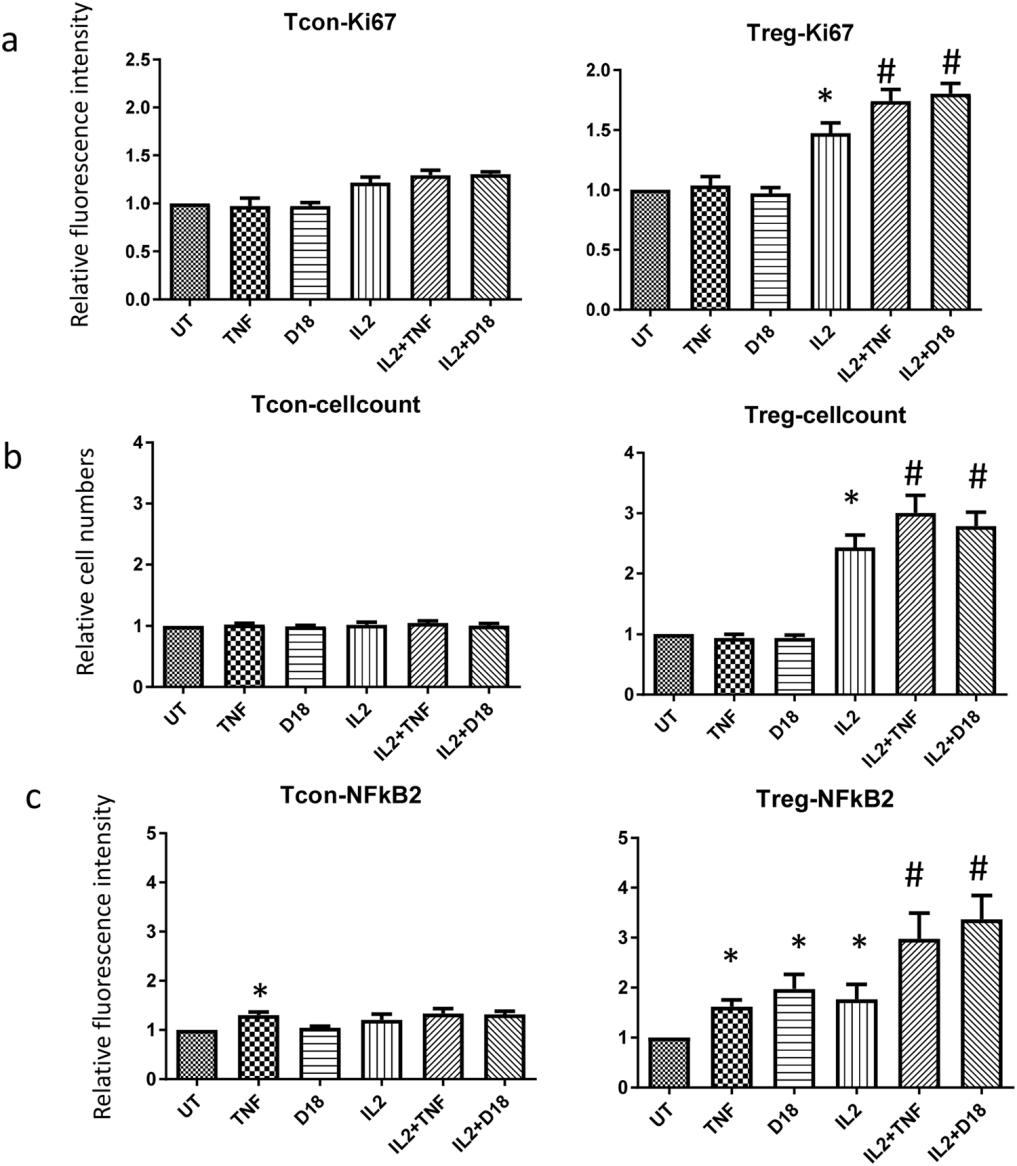
D18 or TNF synergised with IL2 in T regs proliferation. PBMC were treated for 48 hours with the treatment indicated. (**a**) For T cons, single treatment by IL2 induced 21% more Ki67 expression (n = 4, p = 0.036); TNF or D18 alone induced no significant regulation; the combination of TNF or D18 with IL2 induced no significant difference in the level of Ki67 compared to IL2 alone. For T regs, single treatment by IL2 induced significantly more Ki67 expression (49.3% more, n = 4 and p = 0.012); TNF or D18 alone induced no significant regulation of Ki67; the combination of TNF or D18 with IL2 induced significantly more Ki67 expression than IL2 itself (n = 4, p = 0.002 or p = 0.028 respectively). (**b**) A quarter of one million PBMCs were counted and analysed with a flow cytometer, in which none of the treatment significantly regulated the number of T cons. For T regs, IL2 treatment induced a 140% increase in cell numbers, the combination of TNF or D18 induced a further increase in cell numbers (57% more or 35% more respectively, n = 6, p = 0.008 or p = 0.007 respectively). (**c**) For T cons, only single treatment by TNF significantly up-regulated NFκB2 expression (n = 6, p = 0.007), IL2 or the combination of IL2 with TNF or D18 induced no significant regulation. For T regs, all of the single treatments by TNF, D18 or IL2 significantly up-regulated NFκB2 expression (n = 6, p = 0.005, p = 0.021 or p = 0.049 respectively); the combination of TNF or D18 with IL2 induced significantly more NFκB2 expression than IL2 alone (n = 6, p = 0.021 or p = 0.008 respectively). (*p < 0.05 compared to untreated; ^#^p < 0.05 compared to IL2 treated.)

Signalling through TNFR2 has been reported to induce cell proliferation^19^. We investigated if D18 can induce T regs proliferation by examining Ki67 expression as a marker of cell cycle entry. We postulated that TNFR2 ligation alone or in combination with other stimulatory cytokines, such as IL2, may alter T regs responses. PBMCs were treated with TNF and D18 alone or in combination with IL2 for 48 hours. As shown in Fig. 4a, neither TNF nor D18 alone had any significant effect on the level of Ki67 expression in T cons and T regs. Whereas IL2 alone induced increased expression of Ki67 in both T cons and T regs, with a significant and more pronounced effect in T regs. The combination of TNF or D18 with IL2 did not induce any further increase of Ki67 in T cons, but in T regs the combination of either TNF or D18 with IL2 resulted in significantly higher levels of Ki67 compared to IL2 alone. This data was further supported by cell count in which 250,000 PBMCs were counted (Fig. 4b**)**. Following treatment with IL2 in combination of TNF or D18 a significantly higher proportion of PBMCs were T regs compared to treatment with IL2 alone.

As illustrated in Fig. 4c, TNF alone induced upregulation of NFκB2 in both T cons and T regs, while D18 upregulated NFκB2 only in T regs, consistent with our RNA-seq and immunoblot data. IL2 alone also upregulated NFκB2 in T cons and T regs, but had a more marked effect on T regs. TNF or D18 in combination with IL2 did not modulate the IL2 response of T cons, but in T regs, the combination of TNF or D18 with IL2 induced significantly higher levels of NFκB2 than IL2 alone.

**Figure 5:**
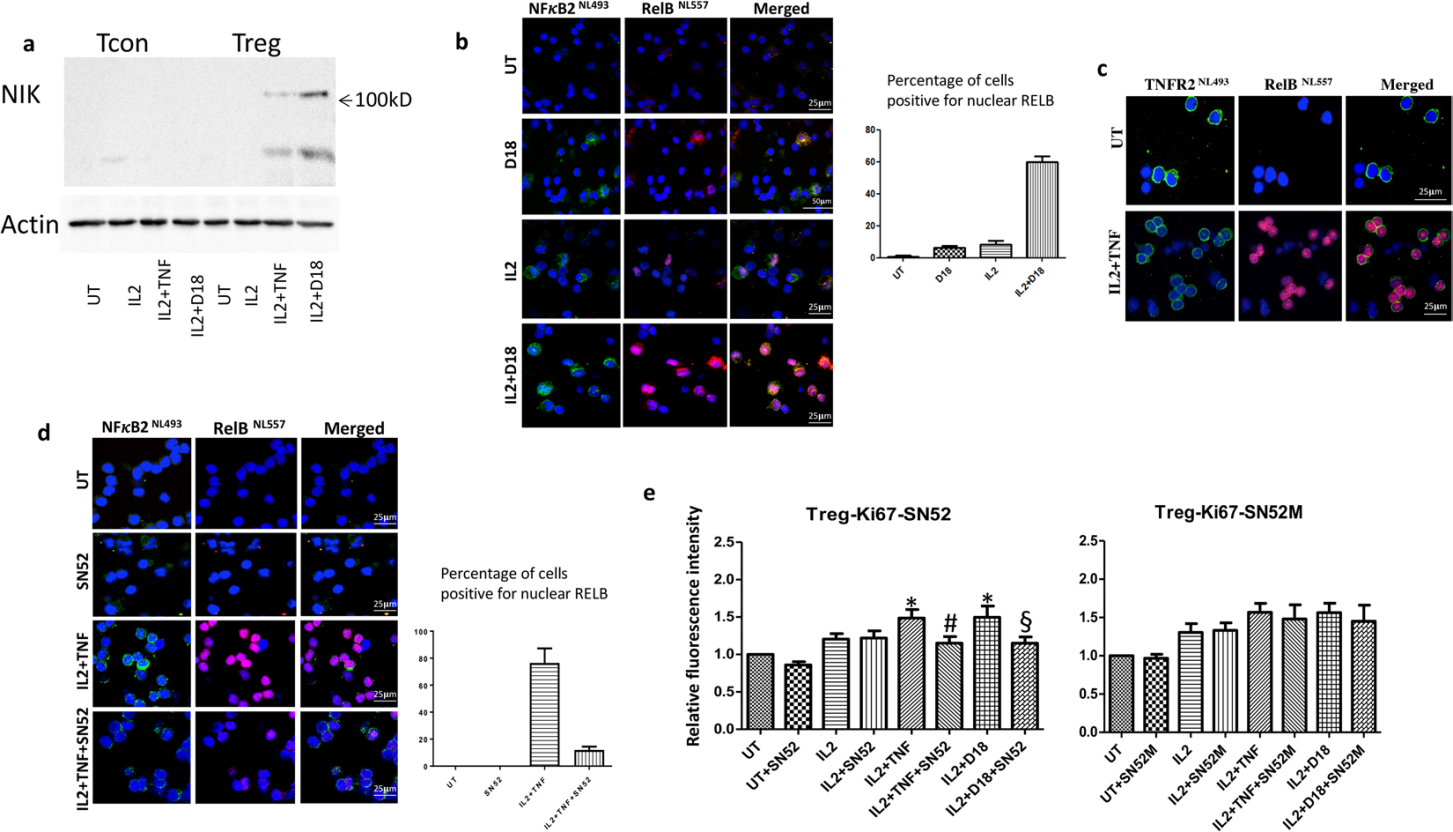
The combination of TNF or D18 with IL2 induced activation of the non-canonical NF-κB pathway in T regs. (**a**) The same number of T cons or T regs were treated with IL2 alone or IL2 plus TNF or D18 for 8 hours. NIK was not detected on T cons by any treatment. In T regs, there was no NIK detectable following treatment with IL2 alone, but the combination treatment of IL2 with TNF or D18 showed a marked expression of NIK. Images were representative of 3 separate experiments. (**b**) T regs were treated with D18 or IL2 or IL2 plus D18 for 8 hours, and cells were immunostained for NFκB2 and RelB. Cells positive for nuclear RelB were counted from 3 different fields for each experiment. Untreated cells showed 0.8 ± 0.8% positive; D18 alone showed 6.1 ± 1.2% positive; IL2 alone showed 8.1 ± 2.6% positive; the combination of IL2 and D18 showed 59.8 ± 3.5% positive. Images are from one of 3 separate experiments with similar results. (**c**) T regs were treated with IL2 plus TNF for 8 hours. Untreated T regs showed strong positivity of TNFR2 in the majority of cells; the combination treatment of IL2 and TNF induced nuclear translocation of RelB in the majority of the cells. The cells that were positive for nuclear RelB were the ones that expressed high levels of TNFR2. (**d**) SN52 was a blocking peptide of the non-canonical NFκB pathway. IL2 plus TNF for 8 hours induced a marked level of nuclear translocation of RelB and NFκB2 in T regs, SN52 pre-treatment blocked this process. (**e**) PBMC were treated with IL2 or the combination of IL2 with TNF or D18 for 48 hours. SN52 alone had no significant effect on Ki67 expression in T regs, nor the IL2 induced upregulation of Ki67 in T regs. However, it significantly reduced the Ki67 level induced by the combination of IL2 and TNF or D18 (n = 4, p = 0.012 or p = 0.032 respectively); whereas the control peptide SN52M had no significant effect. (*p < 0.05 compared to the IL2 untreated; ^#^p < 0.05 compared to IL2 + TNF treated; ^§^p < 0.05 compared to IL2 + D18 treated.)

As the stabilization of NIK is the key event leading to the activation of non-canonical NF-κB pathway^15^, we next examined NIK expression in T regs by immunoblotting. Although all 3 treatments (TNF, D18, IL2) upregulated NFκB2, none of the treatments alone induced detectable NIK stabilization in T regs or T cons (data not shown), whereas the combination of either TNF or D18 with IL2 induced a marked expression of NIK in T regs but not in T cons (Fig. 5a). The activation of non-canonical NF-κB pathway is marked by nuclear translocation of RelB and NFκB2. We next performed immunofluorescence for RelB and NFκB2 on T regs. As shown in Fig. 5b, D18 or IL2 alone induced a relatively small increase of NFκB2 and RelB expression, whereas the combination of IL2 and D18 induced a significant higher number of cells that were positive for nuclear NFκB2 and RelB; although some T regs were still negative after the combination treatment. We then performed immunostaining for TNFR2 and RelB. As shown in Fig. 5c, the combination treatment of IL2 and TNF induced a marked increase of RelB nuclear positive cells; these cells were also TNFR2 positive. In contrast, the TNFR2 negative cells were negative for nuclear RelB. SN52 is a peptide that was shown to block non-canonical NF-κB activation^20^. As shown in Fig. 5d, pre-treatment of itself had no effect on expression of NFκB2 and RelB in T regs, but it reduced the translocation of NFκB2 and RelB in response to the combined treatment of IL2 and TNF. Flow cytometry data in Fig. 5e showed SN52 did not affect IL2 induced Ki67 expression in T regs, but it did prevent the further increase in Ki67 expression induced by IL2 combined with TNF or D18. The control peptide SN52M showed no significant effect. These data suggest that non-canonical NF-κB signalling activated by engagement of TNFR2 on T regs is responsible for the augmentation of IL2-induced proliferation.

### D18 augments T regs expansion and maintain their suppressive function *in vitro*

**Figure 6:**
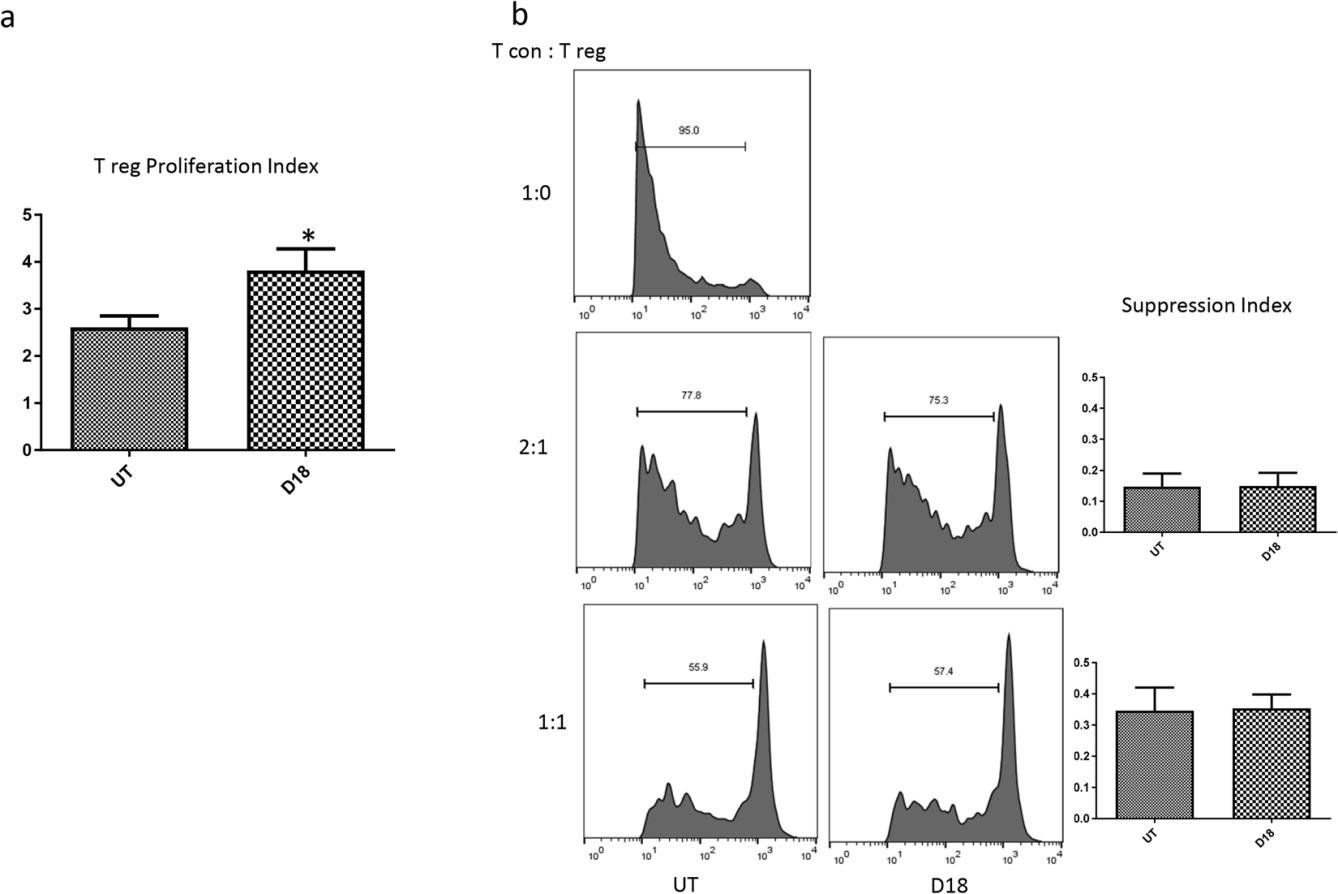
D18 augmented T regs expansion and maintained their suppressive function *in vitro*. (**a**) T regs were expanded for two weeks under two conditions, ‘UT’: with IL2 and anti-CD3 coated Dynabeads; ‘D18’: the UT condition plus D18. Adding D18 to the culture system significantly increased the number of T regs. (*p = 0.031, data were generated from 3 separate experiments). (**b**) A representative suppression assay was shown. T regs expanded under the two conditions showed the same capacity to suppress T cons proliferation under two different cell to cell ratios. Data were generated from 3 separate experiments.

T regs were expanded *in vitro* with either standard combination of IL2 and anti-CD3 coated beads or with addition of D18 to this combination. Adding D18 significantly increased cell number by 46 percent (Fig. 6a). However the suppression ability per cell was not altered as the suppression assay showed no difference between cells expanded by the two methods with or without D18 (Fig. 6b)

## Discussion

Previous studies have indicated a cell intrinsic role of non-canonical NF-κB pathway in maintaining peripheral T regs numbers in mice^21,22^, however the mechanism of this observation and its relevance to human T regs were not clear. Our study showed ligation of TNFR2 by D18 in combination with IL2, selectively activated the non-canonical NF-κB pathway and induced cell proliferation in T regs. In the PBMC culture system, the combination of IL2 and TNFR2 ligation by D18 led to a two fold increase in the relative number of T regs, whereas the number of T cons remained the same. As membrane TNF is the primary physiological ligand for TNFR2^23,24^, our results support a favourable niche for T regs proliferation over T cons around cells that express membrane TNF, consistent with previous report that TNF promotes the function of T regs. Adding D18 to the *in vitro* expansion system of T regs increased cell proliferation, as also shown by previous researchers with TNFR2 antibodies^25^, indicating that TNFR2 ligation plays an important role in regulating T regs proliferation; in contrast to this report the ability to suppress per cell was not altered by D18, which is consistent with the most recent published research^8^. Furthermore, an *in vivo* model using a TNFR2 deficient mouse showed a reduced number but normal function of T regs^12^, which complements our results. However, mutated TNF selective TNFR2 agonists may differ in how they expand T regs. Some TNF agonists proliferate T regs but do not increase TNFR2 expression; some may use different signaling pathways.

The two TNFR2 SNPs rs 522807 and rs 1061622 have been shown to be associated with LPS tolerance^17^ and the effect of anit-TNF treatment^18^ respectively. Data from cells grouped by the two SNPs without manipulating TNFR2 expression by transfection or knock-down showed that the level of TNFR2 cell surface expression was directly related to the strength of its signalling in IκBα degradation, consistent with our previous report and confirming the binding specificity of D18. The IκBα degradation in response to TNF was less in T regs as compared to other T cell groups, in line with a previous report that TNF preferably signals inflammation via TNFR1^9^. We also examined cell surface TNFR1 in our system that showed T regs expressed lower level of TNFR1 compared to Tcons (data not shown). Although, TNF is more potent than D18 in inducing IκBα degradation in T regs (39% vs 27%), considering the different phenotypic subsets of the whole PBMC, TNF signalled the least in T regs while D18 signalled the most in T regs. This is of particular interest for any therapeutic development targeting T regs.

In this study we have carried out RNA sequencing on human T regs with higher purity under flow cytometry CD4^+^CD25hiCD127lo gating^2,26^. The differential analysis of the 3 samples paired by TNF or D18 demonstrated significant transcriptomic alterations by these two treatments. Consistent with the IκBα degradation data, both molecules regulated *NFKB1A* gene in the canonical NF-κB pathway. They also both regulated two components critical in the non-canonical NF-κB pathway in T regs. These results were consistent with previous reports that activation of TNFR2 signalled similar spectrum of pathways to TNF depending on the cellular context^24^. The regulations at gene level were confirmed at protein level with immunoblot and flow cytometry. D18 up-regulated NFκB2 and RelB specifically in T regs. Although the two protein components were up-regulated, the critical step of non-canonical NF-κB pathway activation was the stabilization of NIK^15^. Neither TNF nor D18 alone induced a detectable level of NIK in Tcons and T regs, which might explain why neither of these two treatments induced significant proliferation.

IL2 is a central regulator of T regs function. IL2 selectively induced more Ki67 expression in T regs as compared to T cons in our study. IL2 alone induced a similar degree of NFκB2 up-regulation as TNF or D18 alone, without affecting NIK level thus any detectable non-canonical NF-κB pathway activation. However, a combination of IL2 and TNF or D18 induced an additive effect on NFκB2 and an additive effect on Ki67 expression and NIK stabilization only in T regs. The activation of non-canonical NF-κB pathway was further supported by immunofluorescence that showed nuclear translocation of NFκB2 and RelB protein. The selective determining factor for non-canonical pathway activation was the high level of TNFR2, as only the cells with a high level of TNFR2 showed nuclear translocation of RelB. The activation of this pathway was persistent as shown in supplement figure 3. This result was consistent with a report that TNFR2 activation protected acute graft-versus-host disease in mice by promoting proliferation of T regs^27^. However our result showed TNFR2 activation by D18 alone was not enough to sufficiently activate the non-canonical NF-κB pathway and induce proliferation but rather as a synergetic factor to IL2 to promote T regs proliferation.

As the crucial part of IL2 function is not the presence or absence of IL2R signalling but the graded signalling thresholds, with significantly high level of TNFR2 on the surface, the synergetic effect of TNFR2 ligation may play important role in fine-tuning IL2 signalling in human T regs through non-canonical NF-κB pathway. Our result showed a cell intrinsic pathway in human T regs proliferation in contrast to other T cell population.

## Materials and Methods

### Materials

Healthy donor blood samples were obtained from the NIHR Cambridge BioResource and leukapheresis samples from the UK National Health Service Blood and Transfusion services (NHSBT, Cambridge) with the written informed consent of donors and approval of the local ethics committee-NRES Committee East of England. Monoclonal mouse FITC-, Alexa Fluor 700-, APC/Cy7-, Pacific Blue-, APC-, PE/Cy7-conjugated against human TCR (IP26), CD4 (OKT4), CD8 (SK1) and CD56 (HCD56) (Biolegend, London, UK), CD25 (2A3; BD Pharmingen, Oxford, UK), CD127 (eBioRDR5; eBioscience, Paisley, UK). Mouse PE-conjugated anti-human IκBα (L35A5), anti-human Ki67 (D3B5), anti-human P100/P52NFkB (NFκB2, 18D10)) and isotype control; Rabbit anti-human NIK, RelB, P100/p52NFkB, p-NFkB2p100 and FoxP3 (Cell signalling technology, Hitchin, UK). Introprep (Beckman Coulter, High Wycombe, UK). BD CellFix, Monoclonal mouse PE-conjugated against human CD120b (C83) and isotype control, mouse biotinylated antibody against human CD120a (MABTNFR1-B1) and isotype control, PE streptavidin (BD Biosciences, Oxford, UK). Human recombinant TNF-α and another monoclonal mouse PE-conjugated against human CD120b (22235) (R&D system, Abingdon, UK). SN52 peptide and a control mutated peptide SN52M (Celtek Bioscience, Franklin, USA). Proteinase inhibitor cocktail (Roche Diagnostics Ltd, West Sussex, UK). Dynabeads Human T reg Expander and CFSE kits (Thermofisher Scientific, Loughborough, UK). Unless otherwise indicated, all reagents were from Sigma-Aldrich Company Ltd (Dorset, UK).

### Methods

All methods were performed in accordance with the relevant guidelines and regulations. Peripheral blood mononuclear cells (PBMCs) were isolated from blood by polysucrose density gradient centrifugation (Ficoll-Paque, GE Healthcare Life Sciences, UK). Cell surface staining was performed as previously described^28^. The cells were stained with a surface marker panel plus either control antibodies or anti-CD120b for 15 min at room temperature. Cells were then washed and fixed in BD CellFix and analysed promptly using a BD Fortessa flow cytometer (BD Biosciences, Oxford, UK).

### Flow cytometry

IκBα degradation assay: Cryopreserved PBMCs were thawed in a 37 °C water bath and suspended RPMI1640 + 10% human AB serum and cultured overnight. Cells washed and incubated at 10^6^ cells per ml in 500 µl media per well, supplemented where appropriate with TNF-α (10 ng/ml) or D18 (1 µg/ml) for 15 min at 37 °C/5% CO_2_. Cells were washed and stained with surface markers and fixed/permeabilised according to the manufacturer’s instruction. Then cells were stained with PE-conjugated control antibody or IκBα for 15 min at room temperature^16^. Cells were washed, fixed and analysed promptly using a BD Fortessa flow cytometer.

NFκB2 and Ki67 assay: PBMC were treated with TNF-α (10 ng/ml), or D18 (1 µg/ml), or IL2 (500 units/ml) either alone or in combination for 48 h. For blocking peptide assay, cells were treated with SN52 or SN52M (50 µM/ml) for 30 min prior to the above treatment. Cells were washed and stained with surface markers and fixed/permeabilised according to the manufacturer’s instruction. Then cells were stained with PE-conjugated control antibody or antibody to NFκB2 or Ki67 for 15 min at room temperature. Cells were washed, fixed and analysed promptly using a BD Fortessa flow cytometer.

### Stranded mRNA-Seq

CD4+CD25 + CD127− Tregs were isolated using a T reg isolation kit (Miltenyi Biotec, Surrey, UK), and cell purity was checked with the panel of surface markers described above. T regs were cultured in RPMI1640 supplemented with 10% human AB serum at 37 °C/5% CO_2_ overnight before treatment with TNF (10 ng/ml) or D18 (1ug/ml) for 6 h. Total RNA was collected using RNeasy Plus Micro Kit (Qiagen, Manchester, UK).

Sample sequencing libraries were prepared from 250 ng of total RNA using the TruSeq Stranded mRNA HT sample preparation kit (Illumina, Chesterford UK) according to the manufacturer’s instructions. Samples were individually indexed for pooling using a single index strategy. Libraries were quantified on a Bioanalyzer High Sensitivity DNA1000 chip (Agilent, Cheadle UK) and by qPCR using an NGS Library Quantification Kit (KAPA Biosystems, London UK) on an iCycler qPCR system (Bio-rad, Hemel Hempstead UK). Libraries were then normalised, pooled, diluted and denatured for sequencing on the NextSeq. 500 (Illumina) according to the manufacturer’s instructions. Samples were pooled such that a minimum of 25 M unique clusters per sample was achieved. PhiX control library (Illumina) was spiked into the main library pool at 1% v/v for quality control purposes. Sequencing was performed using a high output flow cell with 2 × 75 cycles of sequencing providing 800 M paired end reads from 400 M unique clusters.

The number of reads that were generated and used at the starting point of the analysis ranged from 27.5 to 33.5 million for each sample, and the range of sequence length was from 35 to 76 for all the samples. Reads were trimmed using TrimGalore v0.3.7 and mapped using STAR v2.4.0 h. Ensembl Homo_sapiens.GRCh38.dna.primary_assembly reference genome file was used to do the mapping of reads, using the annotated transcripts from the Ensembl Homo_sapiens.GRCh38.80 GTF file. The number of reads that map to a genomic feature was calculated using HTSeq v0.6.0 at gene level. Counting of mapped reads at isoform level was performed using RSEM v1.2.22. EdgeR computes effective library sizes using TMM normalization. The normalization factors account for sequencing depth and RNA composition.

### Immunoblotting

Equal number of T regs and the CD4 enriched fraction isolated from the kit described above were cultured overnight before treatment with TNF-α (10 ng/ml), or D18 (1 µg/ml), or IL2 (500 units/ml) alone or in combination for 8 hours. Cells were collected and washed once with ice cold PBS before lysed in lysis buffer (62.5 mM Tris, 2% SDS, 10% glycerol, 10 mM sodium orthovanadate, 10 mM sodium fluoride, proteinase inhibitor cocktail). Samples were boiled in sample buffer (75 mM Tris/HCl, 10% sucrose, 0.2 mg/ml bromophenol blue, 2% SDS) for 5 minutes before separated by SDS polyacrylamide gel electrophoresis and then transferred to nitrocellulose membrane and immunoblotted. Signals were detected by enhanced chemiluminesence using ECL according to the manufacturer’s instructions (Thermo Scienific, Paisley, UK). Images were collected and analysed using Image Lab (Bio-Rad, Hemel Hempsted, UK).

### Immunocytochemistry

Human T regs isolated and cultured overnight as described above were treated with TNF-α (10 ng/ml), or D18 (1 µg/ml), or IL2 (500 units/ml) alone or in combination for 8 h. For blocking peptide assay, cells were treated with SN52 or SN52M (50 µM/ml) for 30 min prior to the above treatment. Cytospins of human T regs were co-immunostained using our previously described protocol^29^. In brief, cells were incubated in cold methanol at −20 °C for 8 min, then rinsed in MilliQ water and in phosphate buffered saline containing 0.01% Tween-20 (PBST). Cells were incubated in blocking buffer (10% fetal inactivated serum in PBST; FCS/PBST) to block non-specific antigen binding for 1 h at room temperature. This was followed by incubation in primary antibody; mouse monoclonal anti-human RelB (D4; sc-48366; Santa Cruz Biotechnology, Middlesex, UK) and rabbit anti-human NFκB2 (15503-1-AP, Proteintech, Manchester, UK) at 1:50 dilution each or goat anti-human TNFR2 (AB-226-PB, R&D Systems, Science Park Abingdon, UK) at 1:200 dilution in FCS/PBST overnight at 4 °C. After several washes in PBST, cells were incubated with rabbit anti-mouse or goat-anti-rabbit IgG affinity purified antibody followed by anti-rabbit-NL557 and anti-goat-NL493 for detection of RelB and NFκB2. For detection of RelB and TNFR2, cells were incubated with rabbit anti-mouse IgG and donkey anti-goat biotinylated antibody followed by anti-rabbit-NL^557^ and Streptavidin-NL^493^ all at 1:100 dilutions in FCS/PBST containing 1ug/mL Hoechst 33342 (ThemoFisher Scientific, Loughborough UK). For a negative control, the primary antibody was omitted. Cells were mounted in VECTASHIELD Mounting Media (Vector Laboratories Ltd., Peterborough, UK) and examined using a Leica TCSPE confocal laser scanning microscope (Leica Microsystems, Milton Keynes, UK). RelB nuclear positive cells were counted in 4 different fields of view at 40x Magnification and cells that showed at least 50% of stained nucleus were counted. Percentage was calculated as the ratio between RelB positive nuclei and total nuclei.

### T regs *in vitro* expansion and suppression assay

T regs were isolated with the above-mentioned kit. 1 × 10^5^ cells per well were cultured in 96-well-round-bottom plates. Cells were either cultured in the media containing Dynabeads for human T reg Expander at a beads-to-cell ratio of 1 to 1 and IL2 at 500 units/ml (untreated); or cultured in the same media plus D18 at 1 ug/ml (D18 treated). Cells were expanded for 2 weeks under the two conditions, and then the Dynabeads were removed. Cells were washed with fresh media and counted. Proliferation index was calculated as the ratio between the increased cell number and basal cell number.

Autologous T cons cryopreserved were thawed the day before and rested overnight. T cons were stained with CFSE following the manufacturer’s instruction. 5 × 10^4^ labelled T cons and expanded T regs were mixed at 2 different ratios and stimulated with the Dynabeads at cell-to-beads ratio of 2 to 1. Cells were collected and analysed after 5 days. Suppression index was calculated as the ratio between decreased percentage of proliferation of T cons and the total percentage of proliferation without T regs.

### Data analysis

Flow cytometry data were analysed using FlowJo (Tree Star, USA). Compensation controls were generated using CompBeads (BD Biosciences). Graphs and statistics were generated using GraphPad Prism software. Results were presented as mean ± s.e.m.as indicated. Differences between two groups were compared using two-tailed student’s t-test.

## Electronic supplementary material

Supplementary Information

## Data Availability

The datasets generated during and analysed during the current study are available from the corresponding author on reasonable request.
