# Supplementary material for: TNFR2 ligation in human T regulatory cells enhances IL2-induced cell proliferation through the non-canonical NF-κB pathway
Source: Sci Rep. 2018 Aug 13;8:12079. doi: 10.1038/s41598-018-30621-4 (PMC6089958; doi:10.1038/s41598-018-30621-4)
Supplement: Supplementary file 1 — Supplementary Information [file 41598_2018_30621_MOESM1_ESM.pdf]

TNFR2 ligation in human T regulatory cells enhances IL2-induced cell proliferation through the non-canonical NF-κB pathway

Jun Wang*[1]; Ricardo Ferreira[2]; Wanhua Lu[1]; Samatha Farrow[3]; Kate Downes[3]; Lutz Jermutus[4]; Ralph Minter[4]; Rafia S. Al-Lamki[1#]; Jordan S. Pober[5#]; and John R. Bradley[1#].

1. Department of Medicine, NIHR Cambridge Biomedical Research Centre, University of Cambridge, Cambridge, United Kingdom.

2. JDRF/Wellcome Diabetes and Inflammation Laboratory, Wellcome Centre for Human Genetics, University of Oxford.

3. Department of Haematology, University of Cambridge, Cambridge, UK.

4. MedImmune Ltd., Granta Park, Cambridge, CB21 6GH, UK.

5. Department of Immunobiology, Yale University School of Medicine, New Haven, CT, United States.

*corresponding author, E-mail: jw389@cam.ac.uk; or jw270uk@hotmail.co.uk

#senior author

Supplement figure 1: Gating strategy

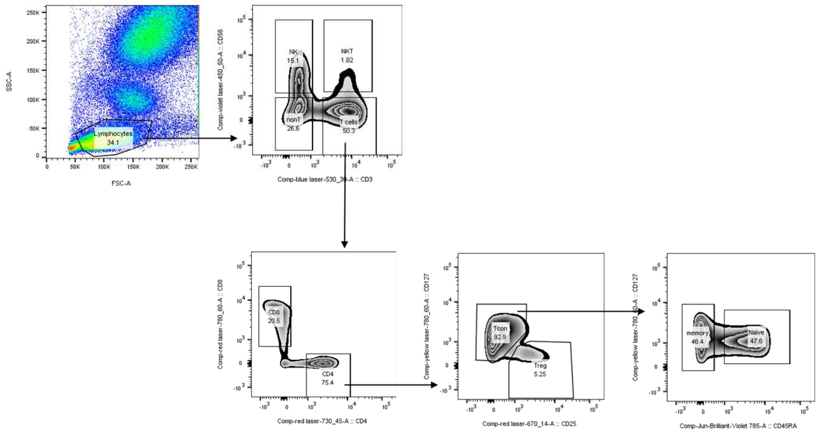

Supplement Figure 2. complete gel image for figure 3 in main article

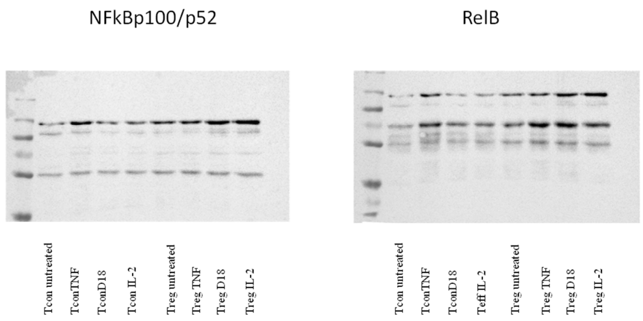

Supplement figure 2. The gel shown in figures were cropped from these two gels with molecular weight marker. The IL-2 treated lanes were cropped out as the results only focused on TNF and D18 treatments.

Supplement figure 3. Phosphorylation of NFkBp100 treated with IL-2 plus TNF

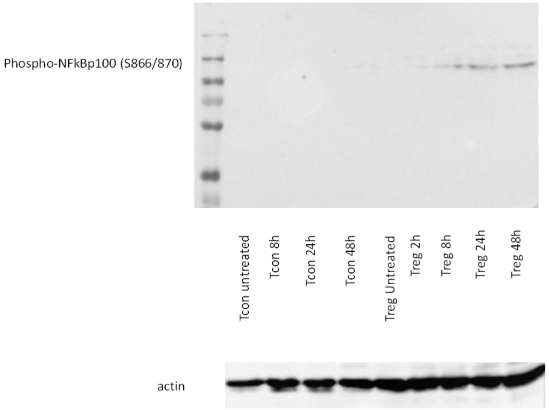

Supplement table 1. Tregs related gene regulation by D18

GeneName	logFC	logCPM	PValue	FDR
FOXP3	-0.049	8.36	0.711932	0.999344
IL2RA	0.008	7.67	0.947081	0.999344
CTLA4	0.083	6.32	0.451433	0.999344
TNFRSF18	0.325	3.50	0.044675	0.831263

Suppement table 2. Tregs related gene regulation by TNF

GeneName	logFC	logCPM	PValue	FDR
FOXP3	-0.034	8.31	0.784063	0.964365
IL2RA	0.030	7.63	0.79942	0.966155
CTLA4	-0.296	6.09	0.027907	0.354443
TNFRSF18	0.796	3.77	5.60E-07	7.53E-05
